# Statin Therapy Negatively Impacts Skeletal Muscle Regeneration and Cutaneous Wound Repair in Type 1 Diabetic Mice

**DOI:** 10.3389/fphys.2017.01088

**Published:** 2017-12-19

**Authors:** Irena A. Rebalka, Andrew W. Cao, Matthew J. Raleigh, Brandyn D. Henriksbo, Samantha K. Coleman, Jonathan D. Schertzer, Thomas J. Hawke

**Affiliations:** ^1^Department of Pathology and Molecular Medicine, McMaster University, Hamilton, ON, Canada; ^2^Department of Biochemistry and Biomedical Sciences and Farncombe Family Digestive Health Research Institute, McMaster University, Hamilton, ON, Canada

**Keywords:** Fluvastatin, streptozotocin, diabetes, diabetic wound healing, diabetic myopathy, PAI-1

## Abstract

Those with diabetes invariably develop complications including cardiovascular disease (CVD). To reduce their CVD risk, diabetics are generally prescribed cholesterol-lowering 3-hydroxy-methylglutaryl coenzyme A reductase inhibitors (i.e., statins). Statins inhibit cholesterol biosynthesis, but also reduce the synthesis of a number of mevalonate pathway intermediates, leading to several cholesterol-independent effects. One of the pleiotropic effects of statins is the reduction of the anti-fibrinolytic hormone plasminogen activator inhibitor-1 (PAI-1). We have previously demonstrated that a PAI-1 specific inhibitor alleviated diabetes-induced delays in skin and muscle repair. Here we tested if statin administration, through its pleiotropic effects on PAI-1, could improve skin and muscle repair in a diabetic rodent model. Six weeks after diabetes onset, adult male streptozotocin-induced diabetic (STZ), and WT mice were assigned to receive control chow or a diet enriched with 600 mg/kg Fluvastatin. Tibialis anterior muscles were injured via Cardiotoxin injection to induce skeletal muscle injury. Punch biopsies were administered on the dorsal scapular region to induce injury of skin. Twenty-four days after the onset of statin therapy (10 days post-injury), tissues were harvested and analyzed. PAI-1 levels were attenuated in statin-treated diabetic tissue when compared to control-treated tissue, however no differences were observed in non-diabetic tissue as a result of treatment. Muscle and skin repair were significantly attenuated in Fluvastatin-treated STZ-diabetic mice as demonstrated by larger wound areas, less mature granulation tissue, and an increased presence of smaller regenerating muscle fibers. Despite attenuating PAI-1 levels in diabetic tissue, Fluvastatin treatment impaired cutaneous healing and skeletal muscle repair in STZ-diabetic mice.

## Introduction

Individuals with Type 1 Diabetes (T1D) are at a significantly elevated risk of developing atherosclerotic cardiovascular disease (CVD), and the risk of death from CVD is 1.7 times greater in diabetic individuals than their age-matched non-diabetic counterparts (Centers for Disease Control and Prevention (CDC), 2014). In 2011, 47% of diabetic patients were reported to have CVD, a statistic that has not changed since 1997 (Centers for Disease Control and Prevention (CDC), 2014). In 2014, this information led to the release of guidelines by the American College of Cardiology and the American Heart Association recommending that all diabetic individuals above the age of 40 be prescribed statins, regardless of the presence of other atherosclerotic CVD risk factors (Stone et al., 2013). As such, in 2014, 63% of adults diagnosed with diabetes were prescribed statins, and this number continues to rise (Gu et al., 2014).

Statins are a class of drugs that act to lower blood cholesterol levels by limiting the endogenous production of cholesterol and promoting clearance of cholesterol-containing lipoproteins though the inhibition of HMG Co-A reductase. This class of lipid-lowering agents has been proven as a key player in the primary and secondary prevention of atherosclerotic CVD events (Sacks et al., 1996; Peto et al., 2002). Evidence has suggested that statins may have additional beneficial properties independent of lowering blood cholesterol. Indeed, the idea of the pleiotropic effects of statins has created quite a large and engaged discussion (Farmer, 2000; Liao and Laufs, 2005; Zhou and Liao, 2010). One of the pleiotropic effects of statins is the inhibition of plasminogen activator inhibitor-1 (PAI-1, also called SERPINE1) transcription via inhibition of the RhoA/ROCK axis (Bourcier and Libby, 2000; Mussoni et al., 2000; Sato et al., 2008; Ni et al., 2013; Sahebkar et al., 2016). PAI-1 is a serine protease inhibitor that acts to impede fibrinolysis, cell adhesion and migration, as well as extracellular matrix breakdown through the inhibition of tissue plasminogen activator and urokinase plasminogen activator (tPA and uPA, respectively) activity (Stefansson and Lawrence, 1996; Kjøller et al., 1997; Li et al., 2003; Dellas and Loskutoff, 2005; Falanga, 2005).

Numerous studies have reported elevated serum and tissue levels of PAI-1 in diabetic individuals (Oishi, 2009; Krause et al., 2011a; Rebalka et al., 2015), and recent studies from our lab have identified PAI-1 as a key component of delayed skeletal muscle and skin repair in T1D rodent models (Krause et al., 2011a; Rebalka et al., 2015). Furthermore, therapeutic strategies to reduce PAI-1 were effective in improving muscle regeneration and dermal wound closure in these animal models (Krause et al., 2011a; Rebalka et al., 2015). Thus, it was the purpose of this study to determine if statin therapy, through its pleiotropic actions on PAI-1, would restore skin and muscle repair in a T1D rodent model. Our findings indicate that although statin-treated T1D tissue displayed decreases in PAI-1 levels, these reductions alone were insufficient to attenuate the impaired muscle and skin regeneration that characterizes those with T1D. In fact, in the current investigation, statin therapy impaired muscle regeneration and cutaneous wound healing to a greater extent than that observed in diabetic rodents.

## Materials and methods

### Animal handling

Male C57BL6/J mice (Jackson Laboratories; Bar Harbor, ME) were provided enrichment material, chow and water *ad libitum*. Animal housing conditions were maintained at 21°C, 50% humidity and a 12 h/12 h light-dark cycle. Experimentation was approved by the McMaster University Animal Research Ethics Board, in accordance with the Canadian Council for Animal Care guidelines. At 10–12 weeks of age, animals were randomly assigned into control (WT) or Streptozotocin diabetic (STZ) groups, and Streptozotocin (one 150 mg/kg injection dissolved in sodium citrate buffer, pH 4.5; Calbiochem; Gibbstown, NJ) was administered to the STZ group. Four weeks after diabetes onset (blood glucose > 14 mM), STZ and WT mice were randomly assigned to receive diet enriched with 600 mg/kg Fluvastatin or control chow (D12081101, D12450K respectively; OpenSource Diets; New Brunswick, NJ). Six weeks after the onset of diabetes, and 2 weeks after the start of diet administration, animals were injured. In order to induce skeletal muscle damage, 10 μM Cardiotoxin (Latoxan; Valence, France) was injected into one tibialis anterior (TA) muscle, as previously reported (Hawke et al., 2007). In order to induce cutaneous damage, each animal received two 6-mm-diameter full-thickness wounds via punch biopsy (Miltex; York, PA) in their dorsal scapular region, as previously reported (Rebalka et al., 2015). Ten days post-injury (24 days of statin or control diet administration), animals were euthanized via cervical dislocation, and tissues were weighed, collected, and stored appropriately for future analyses.

### Fluvastatin quantification

Fluvastatin was quantified in 50 μl of mouse serum, where Atorvastatin (40 ng/ml) was spiked into collected serum for quantification of loss yield following sample preparation. Acetonitrile was added (3:1) to precipitate proteins out of solution and serum was centrifuged to remove particulates. Samples were nitrogen evaporated and suspended in 70:30 ratio of 10 mM ammonium acetate and 90% Acetonitrile, 10% 10 mM ammonium acetate. Samples were loaded into the Agilent 2100 series HPLC (Agilent Technologies; Santa Clara, CA), separated using an Eclipse XDB-C18 column (3.5 μm, 2.1 × 100 mm) and detected with the Bruker micrOTOF II (Bruker; Billerica, MA). The protocol mirrored that detailed by Agilent Technologies Application Notes by Srividya Kailasam; Determination of fluvastatin in plasma using the Agilent 6410B Triple Quadrupole LC/MS system coupled with the Agilent 1200 Series Rapid Resolution LC system.

### Histochemical and immunofluorescent analysis of tibialis anterior skeletal muscle

Eight micrometers frozen TA muscle sections were stained via embryonic myosin heavy chain [eMHC; signal threshold settings used as the detection method (neat; Abcam; Cambridge, MA)] hematoxylin and eosin (H&E; used to quantify fiber area), F4/80 [used to assess total macrophage content; number of F4/80 positive macrophages per unit area of muscle (1:100; Abcam)] and alkaline phosphatase (used to assess vascular content; signal threshold settings used as the detection method) staining using standard protocols.

### Macroscopic, histochemical, and immunofluorescent analysis of cutaneous tissue

Healing was assessed on a macroscopic level by imaging and quantifying eschar size 0, 2, 4, 6, 8, and 10 days post-wounding as previously described (Rebalka et al., 2015). Six micrometers paraffin embedded sections of skin, taken from the center of the wound, were sectioned and air-dried overnight. Masson's Trichrome staining (to measure wound area and assess granulation tissue), CD31 staining [used to assess vascular content; (1:50; Abcam)], and F4/80 staining [used to assess total macrophage content; number of F4/80 positive macrophages per unit area of muscle (1:100; Abcam)] were conducted using standard protocols.

### Histological classification of cutaneous wounds

All Masson's Trichrome images were assigned arbitrary numbers and examined without knowledge of the treatment or group (i.e., blinded analysis). Epidermal and dermal regeneration, as well as granulation tissue formation were evaluated. Unwounded wound margins were used as comparison for scoring (Table 2). This procedure was adapted from previous literature (Altavilla et al., 2001; Galeano et al., 2003).

### Western blotting

TA muscle homogenates were denatured and boiled for 10 min at 95°C. As it has been reported that statin administration may affect both the transcription of PAI-1 as well as glycosylation (Gray et al., 2000; Siddals et al., 2004), lysates were cooled to room temperature whereupon they were incubated for 3 h at 37°C with 1.5 units of Peptide-N-glycosidase F (PNGaseF) (Sigma-Aldrich) to remove glycans. Enzymatic deglycosylation was terminated after 3 h by boiling samples for 5 min at 95°C. Muscle was then analyzed for protein concentration, separated on acrylamide gels via electrophoresis and transferred to polyvinylidene fluoride membranes as previously described (Hawke et al., 2007). Western blotting was undertaken using an anti-PAI-1 (Abcam) primary antibody, appropriate horseradish peroxidase-conjugated secondary antibody, and visualized with the addition of chemiluminescent reagent (Thermo Scientific; Waltham, MA). Images were acquired (Montreal Biotech Inc; Dorval, QC), and 45 kDa bands were quantified using Photoshop (Adobe; Mountain View, CA) with equal loading confirmed by Amido Black staining (Abcam). As seen in Supplementary Figure 1, deglycosylation of lysates resulted in an increase in intensity of the 45 kDa band for PAI-1. Thus, the 45 kDa band in PNGaseF treated samples was used to quantify PAI-1 protein content, with representative full blot images (PAI-1 and Amido Black stained membranes) available in Supplementary Figure 1.

### Statistics

**Figure 1:**
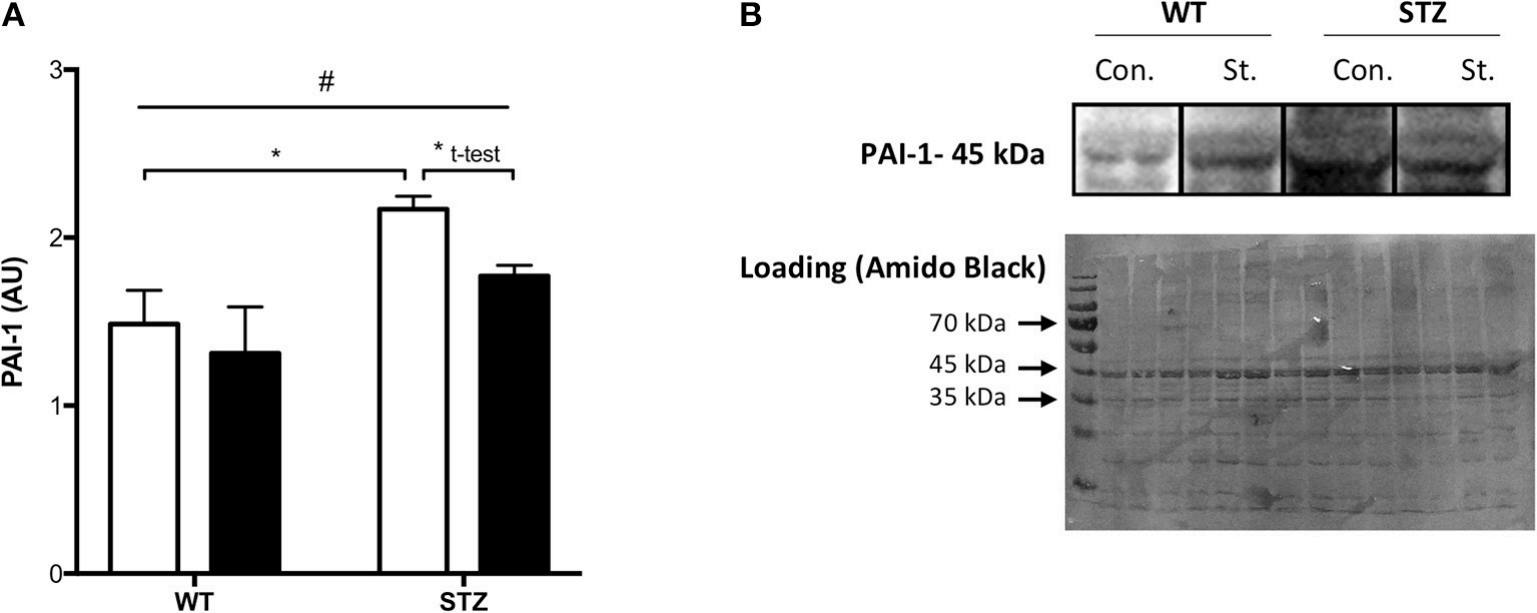
Tissue PAI-1 levels are attenuated by Fluvastatin, but only in the presence of STZ-diabetes. Two-way ANOVA reveals a significant main effect of diabetes (^#^*P* < 0.05) on PAI-1 levels in skeletal muscle **(A)**. An attenuation of PAI-1 content is observed with Fluvastatin treatment, but only in the presence of diabetes. A representative blot is shown in **(B)**. White bars indicate control treatment (Con.). Black bars indicate Fluvastatin treatment (St.). ^*^Indicates significant difference (*P* < 0.05), as determined by Bonferroni's *post-hoc* test following two-way ANOVA. ^*^Indicates a significant difference (*P* < 0.05), as determined by unpaired *t*-test. All data presented as mean ± SEM. *n* = 4–6 for each bar.

All statistical analyses were performed using Prism 7 software (GraphPad; La Jolla, CA). For all analyses, apart from western blotting and eschar analysis, statistical significance was determined using an unpaired *t*-test. Statistical significance for the analysis of western blot bands as well as eschar size were determined using a two-way ANOVA followed by Bonferroni's multiple comparison test. An a priori hypothesis based on available literature was that statin administration would reduce PAI-1 protein expression in diabetic skeletal muscle. In this instance, an unpaired *t*-test was used to specifically compare PAI-1 content between STZ control treatment and STZ with statin treatment (Figure 1). Statistical significance for all methods of investigation was defined as *P* < 0.05. N for each experiment is noted in all figure legends.

## Results

### Fluvastatin content

Serum Fluvastatin analysis revealed that mice fed a control diet had no Fluvastatin in their serum (0 ± 0 μM serum Fluvastatin). A significant increase in serum Fluvastatin content was observed in Fluvastatin-treated groups when compared to control-diet-treated groups (Control diet 0 ± 0 μM serum Fluvastatin vs. Fluvastatin diet 4.463 ± 0.795 μM serum Fluvastatin, *P* = 0.004). No difference in serum Fluvastatin content was observed between WT-Fluvastatin and STZ-Fluvastatin treated animals (WT-Fluvastatin serum 4.268 ± 1.239 μM Fluvastatin vs. STZ-Fluvastatin serum 4.723 ± 1.139 μM Fluvastatin, *P* = 0.402). Fluvastatin content as well as animal information are located in Table 1.

**Table 1:** Animal information and serum Fluvastatin content with SEM.

**Cohort**	**Body weight (g)**	**Blood glucose (mmol/l)**	**Serum Fluvastatin content (μM)**
WT	27.50 ± 0.756	9.95 ± 1.362	0 ± 0
WT Statin	24.82 ± 0.355^*^	10.4 ± 1.111	4.268 ± 1.239^*^
STZ	23.60 ± 0.876^#^	21.65 ± 2.117^#^	0 ± 0
STZ Statin	21.63 ± 0.861^#^	26.03 ± 2.799^#^	4.723 ± 1.139^*^

* *Indicates significant difference relative to respective control*.

# *Indicates significant difference relative to WT group*.

### PAI-1 content

Consistent with the literature, a significant effect of diabetes on muscle PAI-1 expression was observed, namely, an increase in PAI-1 protein expression. Fluvastatin-treated tissue contained less PAI-1 than control treated tissue, but only in the presence of diabetes (Figure 1A). Representative PAI-1 and Amido Black (loading control) blots for these investigations are shown in Figure 1B.

### Cutaneous regeneration

**Figure 2:**
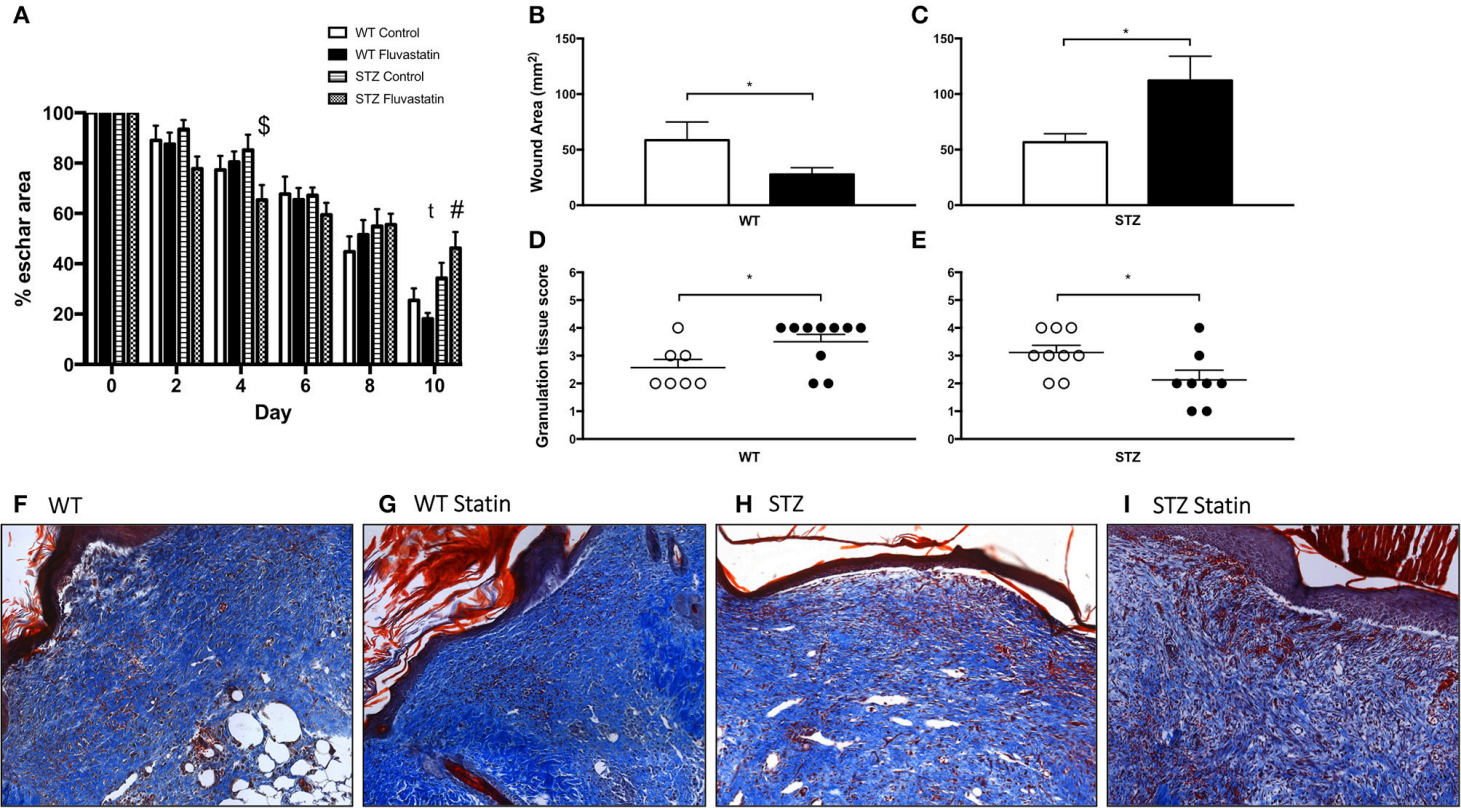
WT cutaneous regeneration is improved by statin treatment, while STZ-diabetic regeneration is impaired by statin treatment. **(A)** Post-wounding improvements in eschar size for all groups are displayed. ^$^Indicates significant difference (*P* < 0.05) between STZ Control and STZ Fluvastatin. ^#^Indicates significant difference (*P* < 0.05) between WT Fluvastatin and STZ Fluvastatin. t indicates trending difference (*P* = 0.08) between STZ Control and STZ Fluvastatin. Fluvastatin administration results in a decrease in wound area in WT wounds **(B)**, whereas the opposite effect is seen in STZ diabetic wounds **(C)**. Similarly, histological assessment of wound healing in WT **(D)** and diabetic **(E)** wounds 10 days after wounding (according to the histological scoring of Table 2) reveal the same effects; an improvement in WT wound repair and a deleterious effect on STZ wound repair with Fluvastatin therapy. **(F–I)** Representative images of wound specimens at 10 days post-wounding are depicted and labeled according to group. White bars **(B,C)** and circles **(D,E)** indicate control treatment. Black bars **(B,C)** and circles **(D,E)** indicate Fluvastatin treatment. ^*^Significant differences (*P* < 0.05) unpaired *t*-test **(B–E)**. All data presented as mean ± SEM. *n* = 10 for each bar in **(A)**, *n* = 10–12 for each bar in **(B,C)**, *n* = 7–10 for each bar in **(D,E)**.

Figure 2A highlights improvements in eschar size from the day of wounding (day 0) to the day of harvest (day 10). Significant improvements in eschar size were observed in STZ-Fluvastatin treated wounds when compared to STZ-control wounds at day 4 post-injury. Ten days post-wounding, STZ-Fluvastatin treated eschar was 28% larger than WT-Fluvastatin treated eschar, suggesting delayed repair in STZ-Fluvastatin treated tissue. To further investigate the effects of Fluvastatin on the subeschar layers of tissue, microscopic histochemical analysis was conducted on excised wound tissue 10 days post-wounding. Wound area analysis of the dermis revealed smaller wounds in WT tissue with Fluvastatin treatment (Figure 2B). Contrastingly, Fluvastatin treatment resulted in larger wound sizes in STZ mice 10-days post-wounding (Figure 2C). Histological scoring of granulation tissue (as per Table 2) mirrored results of the wound area analysis, showing improvements in tissue repair in WT wounds with Fluvastatin treatment (Figure 2D), and an impediment to repair with Fluvastatin treatment in STZ tissue (Figure 2E). Representative wound bed images with Masson's Trichrome staining are shown in Figures 2F–I.

**Table 2:** Histological scores of wounds.

**Scores**	**Epidermal and dermal regeneration**	**Granulation tissue thickness**
1±	Little epidermal and dermal organization. Very thick epidermis. Substantial dermal edema.	Thin granular layer.
2±	Moderate epidermal and dermal organization. Moderate epidermal thickness. Mild dermal edema.	Moderate granular layer.
3±	Complete remodeling of epidermis and dermis. Thin, organized epidermis. Little dermal edema.	Thick granular layer.
4±	Complete remodeling of epidermis and dermis. Epidermis appears as uninjured.	Very thick granulation layer.

### Muscle regeneration

**Figure 3:**
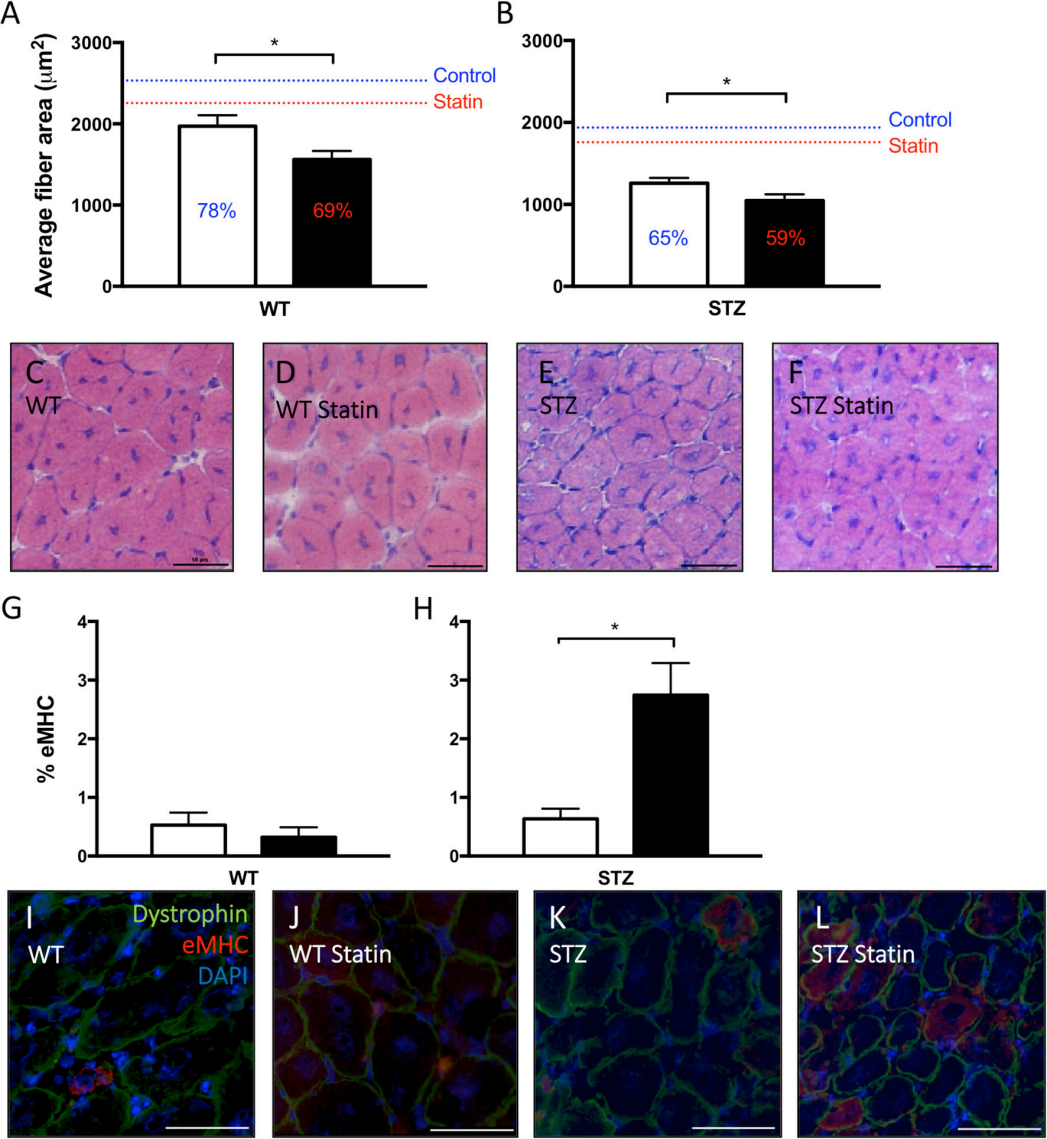
Statin therapy delays STZ-diabetic skeletal muscle regeneration. In both WT **(A)** and STZ **(B)** skeletal muscle, smaller average myofiber area, signifying delayed regeneration, is observed in Fluvastatin treated muscle when compared to the respective control. Percent of uninjured muscle fiber size is displayed on each bar, and mean uninjured fiber sizes are indicated by a line for reference. Representative images depicting fiber size of regenerating myofibers are shown in **(C–F)**, and are labeled by group. Embryonic myosin heavy chain (eMHC), a myosin isoform present early in myofiber development, is elevated in Fluvastatin-treated STZ muscle **(H)**, but not Fluvastatin-treated WT muscle **(G)**, (*P* = 0.26) when compared to its control-treated counterpart. White bars indicate control treatment. Black bars indicate Fluvastatin treatment. **(I–L)** Representative eMHC images, indicating an abundance of eMHC (red staining) in regenerating diabetic statin myofibers, are labeled according to group. ^*^Significant differences (*P* < 0.05), as determined by unpaired *t*-test. All data presented as mean ± SEM. *n* = 4–6 for each bar.

When compared to muscle from control-treated rodents, the cross-sectional area of regenerating fibers was significantly reduced following Fluvastatin treatment in both WT (Figure 3A) and STZ (Figure 3B) muscle, indicating a delay in the regenerative capacity. Representative images are shown in Figures 3C–F. To confirm the suspected delay in skeletal muscle repair, eMHC immunofluorescent analysis was conducted. eMHC is a myosin isoform that is present during the early stages of skeletal muscle regeneration. A greater presence of eMHC was observed in regenerating Fluvastatin-treated STZ muscle (Figure 3H). This effect was rarely seen in WT muscle, with trace amounts of eMHC present in both treatment groups (Figure 3G). This protracted expression of eMHC, which should reach peak expression at 2–3 days post-injury (Schiaffino et al., 2015), supports the conclusion that Fluvastatin treatment delays STZ-diabetic skeletal muscle repair. Representative images are shown in Figures 3I–L.

### Cutaneous vascularity

**Figure 4:**
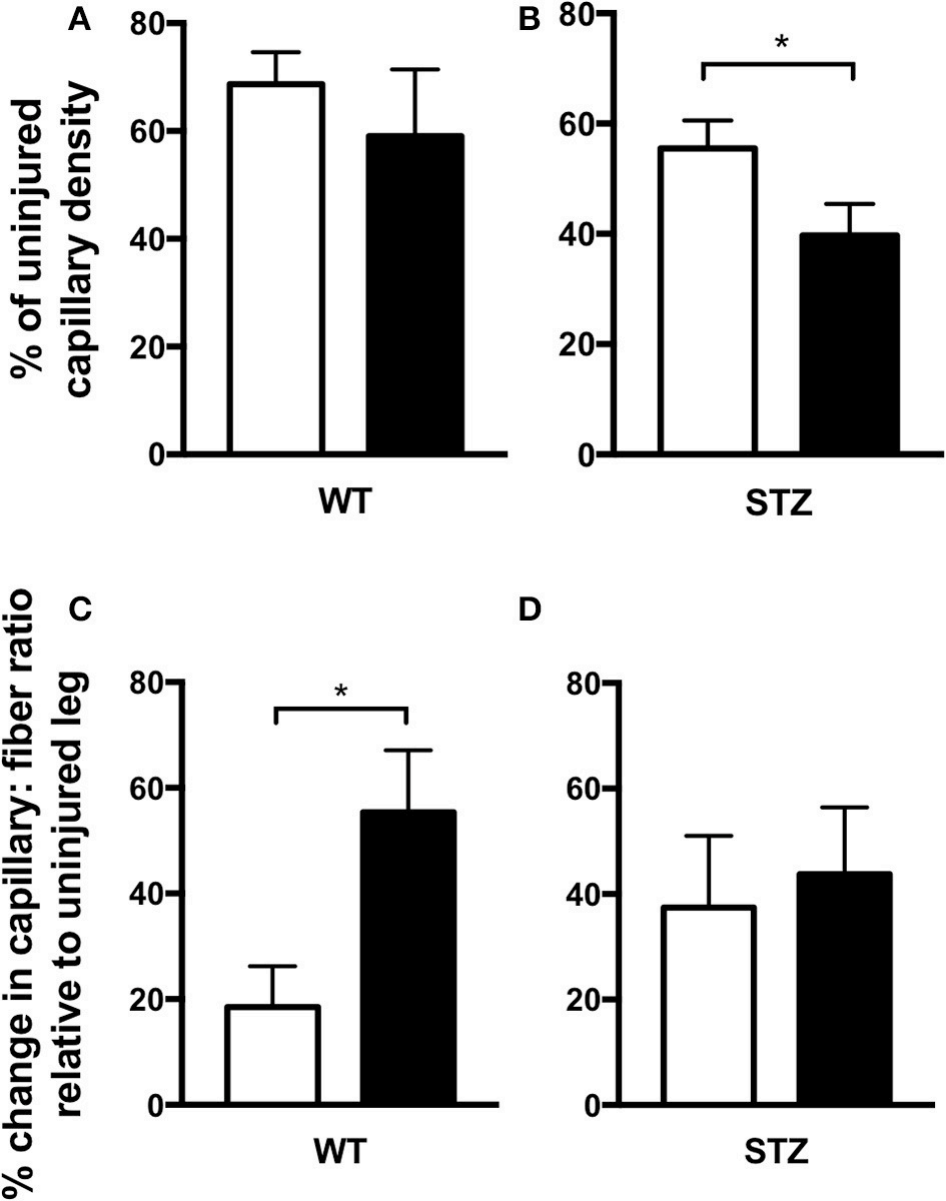
Fluvastatin is deleterious to STZ-diabetic cutaneous vascular regeneration and does not improve diabetic skeletal muscle revascularization. Expression of wound-site vascular content as a percentage of uninjured vascular content uncovers an approximate 60% regeneration of vascularity in both control and Fluvastatin treated WT groups **(A)**. In STZ-diabetic wounds, a 16% decrease in the restoration of vascularity is observed with Fluvastatin treatment when compared to control treatment **(B)**. Expression of changes in capillary: fiber ratio relative to uninjured muscle uncovers an exacerbated increase in vascular perfusion with Fluvastatin administration in WT groups **(C)**. When normalized to the uninjured ratio, vascularity is not improved in regenerating STZ diabetic muscle **(D)**. White bars indicate control treatment. Black bars indicate Fluvastatin treatment. ^*^Significant differences (*P* < 0.05), as determined by unpaired *t*-test. All data presented as mean ± SEM. *n* = 6–10 for each bar in **(A,B)**, *n* = 5–6 for each bar in **(C,D)**.

To assess vascular density within the wound site, immunofluorescent staining for CD31 (PECAM-1), an endothelial cell marker, was conducted (Figure 4). Because statin treatment has been reported to systemically affect angiogenesis (Kureishi et al., 2000; Urbich et al., 2002; Weis et al., 2002; Muck et al., 2004), wound-site vascularity was expressed as a percentage of uninjured vascular density. This measure allows us to glean the effects of Fluvastatin on wound-site vascularity without being skewed by potential confounding effects of pre-injury vascular changes. Vascularity of the wound site was unchanged with Fluvastatin treatment in WT tissue (both at ~60% of their unwounded vascular density, Figure 4A). STZ-diabetic wounds, however, displayed 16% less vascularity with Fluvastatin treatment (Figure 4B).

### Muscle vascularity

To assess vascular density in regenerating areas of skeletal muscle, alkaline phosphatase staining was conducted (Figure 4). Capillary to fiber ratio was investigated, as a higher capillary to fiber ratio allows a greater magnitude of perfusion to each muscle fiber. This comprehensive perfusion can be critical to optimal muscle repair. As in cutaneous repair, vascular density of regenerating muscle was expressed relative to uninjured vascular density to define the effects of Fluvastatin without being skewed by any potential confounding effects of pre-injury vascular changes. Interestingly, capillary to fiber ratio was increased with Fluvastatin treatment in WT tissue (Figure 4C), however, regenerating diabetic muscle displayed no improvements in vascularity with Fluvastatin treatment (Figure 4D).

## Discussion

The majority of individuals diagnosed with diabetes are, or will invariably be, prescribed statins due to their known benefits to reduce CVD risk (Gu et al., 2014). While the cardiovascular benefits of statins in the T1D population are well-known (Peto et al., 2002; Cholesterol Treatment Trialists' (CTT) Collaborators, 2008), this is the first study, to our knowledge, that investigates the effects of statins on changes in PAI-1 for the restoration of tissue repair in T1D. Due to the substantial number of diabetic individuals currently prescribed statins, defining the pleiotropic effects of statins in this population is of utmost importance. These investigations will not only lead to an understanding and reduction of drug side effects, but may also lead to prescription of new or alternate classes of compounds specifically targeting one pleiotropic stream of statin action.

Consistent with literature (Oishi, 2009), an increase in PAI-1 was seen in STZ-diabetic rodents with only 7 weeks of diabetes in the current investigation. Fluvastatin treatment, as expected, significantly attenuated the rise in PAI-1 expression observed with STZ-diabetes. As no differences in circulating Fluvastatin levels were observed between WT-Fluvastatin and STZ-Fluvastatin treated animals (4.268 ± 1.239 and 4.723 ± 1.139 μM Fluvastatin, respectively, *P* = 0.402), a difference in treatment was not responsible for this effect.

Despite the attenuation in PAI-1 levels in STZ-diabetic tissue, impaired regeneration persisted in both the skin and muscle. This was an unexpected finding, as it was hypothesized that mitigating the presence of PAI-1 would increase matrix porosity; facilitating the infiltration of reparative cells into regenerating skin and muscle as previously reported (Krause et al., 2011a; Rebalka et al., 2015). Although it is evident that the primary mechanism of statin action in the current investigation is not via PAI-1 attenuation, the muscle and skin examined in the current investigation appears to display similar reparative profiles and changes with Fluvastatin administration. This seemingly parallel phenotype in both tissue types alludes to an alternative yet possibly equivalent mechanism of action.

To complement our findings of delayed diabetic tissue repair with statin treatment, macrophage quantification was completed. Despite the known anti-inflammatory effects of statins (Jain and Ridker, 2005; Adami et al., 2012), when compared to WT animals receiving statin treatment, STZ-diabetic animals treated with Fluvastatin displayed an overabundance of macrophages in both regenerating skin (Table 3: STZ Fluvastatin 1.87 ± 0.28% total wound area vs. WT Fluvastatin 0.64 ± 0.15, *P* = 0.003) and skeletal muscle (Table 3: STZ Fluvastatin 251.40 ± 53.18/mm^2^ vs. WT Fluvastatin 136.20 ± 17.77, *P* = 0.03). Whereas, a deficiency in macrophage content does not allow for adequate tissue repair, the overabundance of macrophages in the wound site is also detrimental to the healing process, chronically leaving wounds in the early phases of tissue repair (Goren et al., 2007). This increased presence of inflammatory cells further supports our aforementioned results of exacerbated impairments in diabetic repair with Fluvastatin treatment. As alterations in the inflammatory phase of wound repair, including prolonged inflammation, are already present in the diabetic environment (Fahey et al., 1991; Wetzler et al., 2000), further delay of inflammatory clearance with statin administration is less than ideal.

**Table 3:** Macrophage content- normalized to respective control group with SEM.

**Cohort**	**Macrophage content in skin: % total wound area**	**Macrophage content in skeletal muscle: macrophages/mm^2^**
WT Statin	0.64 ± 0.15	136.20 ± 17.77
STZ Statin	1.87 ± 0.28^*^	251.40 ± 53.18^*^

* *Indicates significant difference between WT Statin and STZ Statin groups*.

It has been observed that statin therapy improves cutaneous healing; having anti-inflammatory effects, accelerating tissue repair, and reducing lesion area in non-diabetic subjects (Adami et al., 2012; Suzuki-Banhesse et al., 2015). Indeed, in the current investigation, improved cutaneous repair was observed in non-diabetic rodents. The opposing effect, namely larger wound area and decreased granulation tissue quality, were observed in the presence of diabetes. Due to the transient and dynamic presence of the phases of tissue repair, future extensions of this investigation should focus on exploring the effects of Fluvastatin therapy at various timepoints in the repair process to glean a more comprehensive understanding of the effect of Fluvastatin at each phase in tissue regeneration. Previous reports indicate that all beneficial effects of Atorvastatin, another lipophilic statin, on cutaneous repair are no longer visible after day 7 post-injury (Suzuki-Banhesse et al., 2015). Indeed, in the current investigation, eschar analysis of Fluvastatin-treated STZ rodents revealed a 20% reduction in eschar size at 2 days post-injury, and a significant 30% reduction in eschar size 4 days post-injury when compared to STZ control-treated rodents (Figure 2A). This result foreshadows a positive effect of statins on the underlying tissue at these early timepoints.

Although the effect of statins on the repair of skeletal muscle have yet to be studied extensively, Trapani and colleagues have reported a decrease in myocyte fusion, as well as smaller, thinner myofibers resembling congenital myotonic dystrophy-affected myotubes with statin administration *in vitro* (Trapani et al., 2012). Furthermore, myoblasts treated with statin maintained higher eMHC levels and lower levels of adult myosin isoforms than their vehicle-treated counterparts (Trapani et al., 2012). This representation of delayed myotube maturation and muscle fiber formation is mirrored in the current investigation by the presence of smaller myofibers and a persistence of eMHC in diabetic muscle receiving statin therapy. Interestingly, Trapani and colleagues were able to reverse the deleterious effects of statins *in vitro* via mevalonate therapy; restoring the activity of the HMG CoA reductase pathway (Trapani et al., 2012). Similar to cutaneous repair, skeletal muscle repair is impaired in T1D (Gulati and Swamy, 1991; Vignaud et al., 2007; Krause et al., 2011b, 2013). Given information gleaned from previous work as well as this investigation, it is hypothesized that Fluvastatin is inhibiting myoblast fusion *in vitro* via a mechanism independent of extracellular matrix remodeling. Although further investigation into this mechanism are warranted, the current investigation complements previous reports of the negative effects of statins on skeletal muscle health, which include changes in the adaptive response to stressors (current work), storage, and transport of lipids (Rebalka et al., 2016), and metabolic capacity/glucose handling (Ridker et al., 2008; Duvnjak and Blaslov, 2016).

We know that statins are effective in reducing the occurrence of macrovascular CVD events in diabetic individuals (Peto et al., 2002; Cholesterol Treatment Trialists' (CTT) Collaborators, 2008), but previous reports have implicated statins in impairing microvascular function in patients with T1D (Tehrani et al., 2013). Indeed, a decrease in vascular presence following cutaneous injury was observed in the current investigation. Contrastingly, statins have been shown to upregulate the presence vascular stimulants in the early phases of tissue repair in type 2 diabetic patients (Bitto et al., 2008; Farsaei et al., 2012). Due to the knowledge that statin therapy modifies angiogenesis (Kureishi et al., 2000; Urbich et al., 2002; Weis et al., 2002; Muck et al., 2004), wound vascularity was normalized to pre-injury vascular presence in the current investigation. No other studies, to our knowledge, look at these absolute wound vascular values, and it would be interesting to draw these comparisons from previously published papers. Given the lack of both cardiovascular and microvascular disease in STZ-diabetic rodents, further experimentation using other preclinical models of T1D that more closely mimic the vascular aspects of diabetes may provide further insight into the impact of statin therapy on microcirculation and its effects on tissue repair. This would allow us to more accurately extrapolate our findings to the clinical diabetic population.

Overall, although Fluvastatin was shown to attenuate PAI-1 levels in diabetic tissue, impaired cutaneous and skeletal muscle regeneration persisted. In contrast to our hypothesis, the use of statin therapy as a means to inhibit PAI-1 and alleviate impaired cutaneous and muscle regeneration is likely ineffective in those with T1D. Many diabetic individuals, already exhibiting impaired cutaneous and skeletal muscle regeneration, are prescribed statins. As such, the mechanism resulting in the detrimental effects of Fluvastatin on tissue repair observed in the current investigation warrant further investigation.

## Author contributions

IR, JS, and TH designed the experiments. IR, AC, MR, BH, and SC performed experiments and data analyses. IR and TH wrote the manuscript. All authors revised the manuscript and provided final approval of the version to be published.

### Conflict of interest statement

The authors declare that the research was conducted in the absence of any commercial or financial relationships that could be construed as a potential conflict of interest.

## Abbreviations

CVD: Cardiovascular disease
eMHC: Embryonic myosin heavy chain
HMG CoA: 5-hydroxy-3-methylglutaryl-coenzyme A
PAI-1: Plasminogen activator inhibitor-1
STZ: Streptozotocin
TA: Tibialis anterior
tPA: Tissue plasminogen activator
T1D: Type 1 Diabetes Mellitus
uPA: Urokinase plasminogen activator
WT: Wild-type.
