# Supplementary figures and images for: Statin Therapy Negatively Impacts Skeletal Muscle Regeneration and Cutaneous Wound Repair in Type 1 Diabetic Mice
Source: Front Physiol. 2017 Dec 19;8:1088. doi: 10.3389/fphys.2017.01088 (PMC5742241; doi:10.3389/fphys.2017.01088)

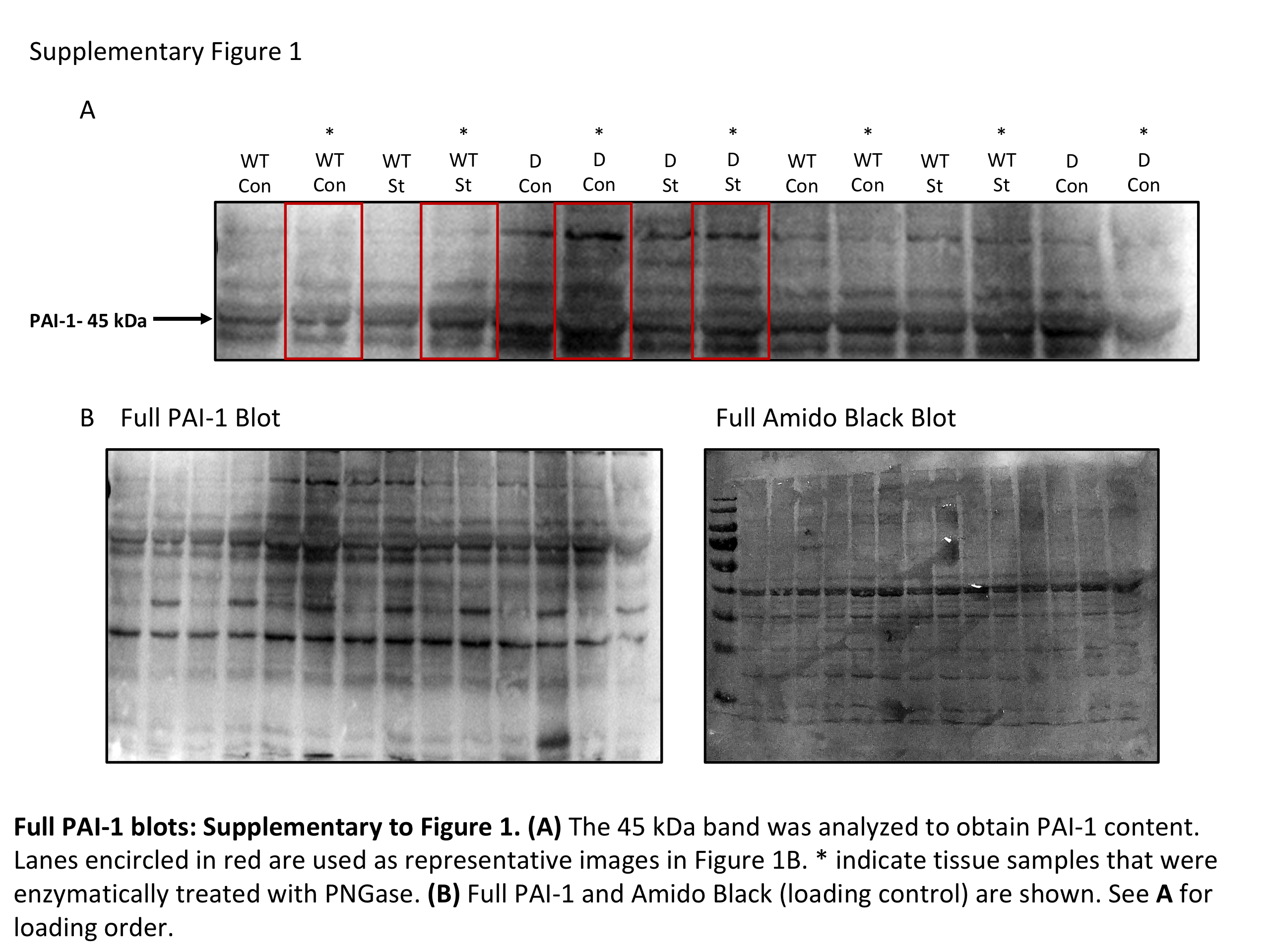

Supplement: Supplementary file 1 [file Image1.tiff]
